# Microbial-derived imidazole propionate links the heart failure-associated microbiome alterations to disease severity

**DOI:** 10.1186/s13073-024-01296-6

**Published:** 2024-02-08

**Authors:** Sajan C. Raju, Antonio Molinaro, Ayodeji Awoyemi, Silje F. Jørgensen, Peder R. Braadland, Andraz Nendl, Ingebjørg Seljeflot, Per M. Ueland, Adrian McCann, Pål Aukrust, Beate Vestad, Cristiane Mayerhofer, Kaspar Broch, Lars Gullestad, Knut T. Lappegård, Bente Halvorsen, Karsten Kristiansen, Johannes R. Hov, Marius Trøseid

**Affiliations:** 1 Research Institute of Internal Medicine, Oslo University Hospital Rikshospitalet, Oslo, Norway; 2 Institute of Clinical Medicine, Faculty of Medicine, University of Oslo, Oslo, Norway; 3 Department of Medicine, Sahlgrenska University Hospital, Gothenburg, Sweden; 4 Department of Transplantation Medicine, Norwegian PSC Research Center, Oslo University Hospital Rikshospitalet, Oslo, Norway; 5 Department of Cardiology, Oslo University Hospital Ullevål, Oslo, Norway; 6 Center for Clinical Heart Research, Oslo University Hospital Ullevål, Oslo, Norway; 7 Section of Clinical Immunology and Infectious Diseases, Oslo University Hospital Rikshospitalet, Oslo, Norway; 8 Bevital, Bergen, Norway; 9 Department of Cardiology, Oslo University Hospital Rikshospitalet, Oslo, Norway; 10 Division of Internal Medicine, Nordlandssykehuset, 8005 Bodø Norway; 11 Institute of Clinical Medicine, University of Tromsø, 9037 Tromsø, Norway; 12 BGI-Shenzhen, Shenzhen, 518083 China; 13 Laboratory of Genomics and Molecular Biomedicine, Department of Biology, University of Copenhagen, 2100 Copenhagen, Denmark; 14 Section of Gastroenterology, Department of Transplantation Medicine, Oslo University Hospital, Oslo, Norway

**Keywords:** Imidazole propionate, Gut microbiota, Inflammation, Heart failure

## Abstract

**Background:**

Interactions between the gut microbiota, diet, and host metabolism contribute to the development of cardiovascular disease, but a firm link between disease-specific gut microbiota alterations and circulating metabolites is lacking.

**Methods:**

We performed shot-gun sequencing on 235 samples from 166 HF patients and 69 healthy control samples. Separate plasma samples from healthy controls (*n* = 53) were used for the comparison of imidazole propionate (ImP) levels. Taxonomy and functional pathways for shotgun sequencing data was assigned using MetaPhlAn3 and HUMAnN3 pipelines.

**Results:**

Here, we show that heart failure (HF) is associated with a specific compositional and functional shift of the gut microbiota that is linked to circulating levels of the microbial histidine-derived metabolite ImP. Circulating ImP levels are elevated in chronic HF patients compared to controls and associated with HF-related gut microbiota alterations. Contrary to the microbiota composition, ImP levels provide insight into etiology and severity of HF and also associate with markers of intestinal permeability and systemic inflammation.

**Conclusions:**

Our findings establish a connection between changes in the gut microbiota, the presence, etiology, and severity of HF, and the gut-microbially produced metabolite ImP. While ImP appears promising as a circulating biomarker reflecting gut dysbiosis related to HF, further studies are essential to demonstrate its causal or contributing role in HF pathogenesis.

**Trial registration:**

NCT02637167, registered December 22, 2015.

**Supplementary Information:**

The online version contains supplementary material available at 10.1186/s13073-024-01296-6.

## Background

The gut microbiota comprises complex bacterial communities whose metabolic activities and interactions with the immune system extend beyond the gut itself. Host-microbiota interactions have been proposed to contribute to the pathogenesis of cardiometabolic diseases such type 2 diabetes (T2D), cardiovascular disease (CVD), and heart failure (HF) [1]. Over the last decade, several studies have reported that the gut microbiota composition and its predicted functional potential differ between subjects with HF and healthy controls [2–5]. A decreased abundance of taxa from the short chain fatty acid (SCFA)-producing *Lachnospiraceae* or *Ruminococcaceae* families is frequently observed. Nonetheless, significant heterogeneity exists in the reported microbiota composition across these studies, which may be attributed to factors such as variations in case mix, diet, sampling procedures, and sequencing techniques.

The gut microbiota produces numerous metabolites that can be absorbed into the systemic circulation [6]. The diet- and microbiota-dependent metabolite trimethylamine-N-oxide (TMAO) [7, 8], microbiota-generated secondary bile acids [9], and, more recently, the microbiota-derived phenylacetylglutamine [10] have all been found elevated in subjects with HF and to associate with various gut microbes. However, clear links between these metabolites and disease-related gut microbiota changes are lacking [11]. The microbially produced metabolite imidazole propionate (ImP), previously linked to T2D [12], was recently discovered to be increased in subjects with HF [13], but the bacterial taxa responsible for the elevated ImP in HF remain unknown.

Here, we characterize the gut microbiota composition in one of the largest HF cohorts to date (*n* = 166) and integrate these findings with targeted measurements of circulating ImP in subjects with and without HF. We expand on previous studies by linking circulating ImP not only to etiology and severity of HF but also to a specific gut compositional shift, altered intestinal permeability, and systemic inflammation, overall establishing a HF-specific connection between gut dysbiosis and the production of a potentially harmful metabolite.

## Methods

### Study participants/cohorts

**Fig. 1:**
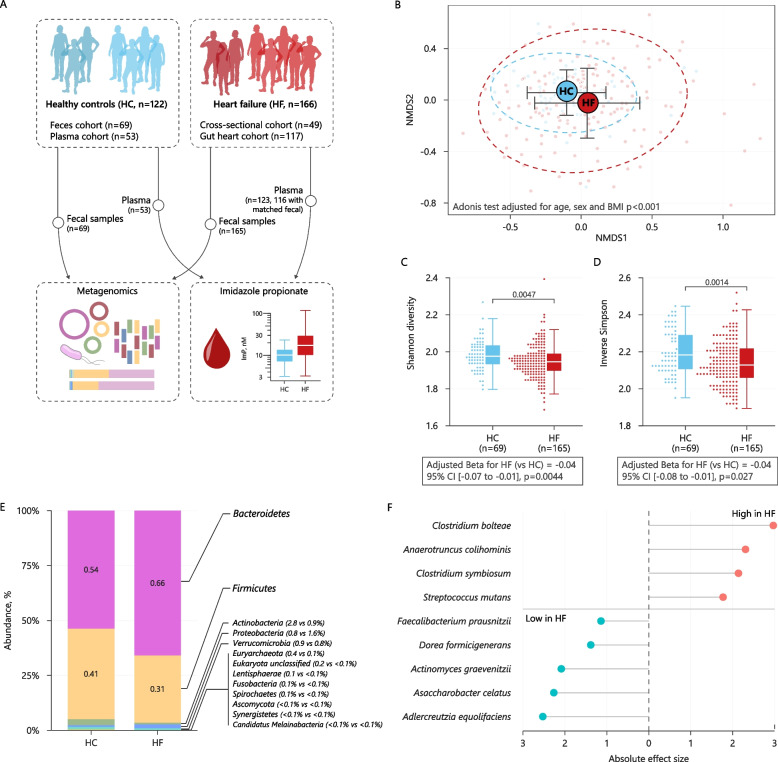
Subjects with heart failure have a specific microbiota compositional shift. **A** Graphical representation of the cohorts used in the study. **B** Principal coordinate analysis of Bray–Curtis dissimilarities obtained for the metagenomic species profiles, by non-metric multi-dimensional scaling (NMDS) plot in heart failure (HF) and healthy controls (HC). Box plots and dot plots of the alpha diversity indices Shannon (**C**) and inverse Simpson (**D**) in HF compared to HC. Differences in distributions of Shannon diversity index and inverse Simpson were calculated using Wilcoxon tests. Multivariable linear regression models were fitted to estimate whether the differences remained after adjusting for age, sex, and BMI (model estimates are shown below the plots). **E** Relative abundance of bacterial phyla in HF and HC. **F** Compositional differences on species levels, displaying effect size from MaAsLin2 modeling, with age, sex, and BMI as covariates, in HF compared to HC (see also Additional file 2 Table S1-3)

Study participants were recruited from two previous cohorts with similar sampling protocols for gut microbiota samples: Norwegian participants from the randomized GutHeart study (*N* = 117) [14] (end points available in Additional file 1) and a cross-sectional study of subjects with chronic HF (*N* = 49) [4] (Fig. 1A). One participant was excluded since BMI was missing. Subjects with HF were eligible for inclusion if they had a stable systolic HF with left ventricular ejection fraction (LVEF) < 40% and New York Heart Association (NYHA) functional class II–III with no changes in medications during the last 3 months prior to inclusion in the study. We included 69 stool samples from healthy controls who were selected based on their disease history which indicated overall good health and no regular medication usage. We also included plasma samples from a separate cohort of 53 apparently healthy individuals (median age 67 years, 57% men), based on disease history and clinical examination, for comparison of ImP levels. No subjects included in the study had used any antibiotics or probiotics in the last 3 months prior to sampling.

### Sample collection, DNA extraction, and shotgun sequencing

Stool samples from all participants were collected in tubes with a DNA-stabilizing solution (Stratec Molecular GMBH, Berlin, Germany). Fecal DNA was extracted using the PSP Spin Stool DNA kit (Stratec Molecular GMBH) following the manufacturer’s protocol, modified by adding a bead-beating step as recommended in a previous study [15]. Briefly, 0.5 g of 0.1 mm zirconia beads and 4 zirconia beads of 3 mm (from the PSP kit) were added to 1.4 mL of homogenized stool for extraction. The samples were treated in a bead beater at room temperature at 5.5 ms for 3 × 60 s, with cooling steps in between. Heat incubation, prelysis, and the rest of the extraction procedure were performed according to the manufacturer’s protocol. All samples were analyzed by shotgun sequencing using 150 bp paired-end sequencing on the MGISEQ-T7 platform.

### Metagenomic sequence data processing

The shotgun metagenomic sequencing generated ~ 12.18 (× 2) billion reads for 235 samples. The samples had a median read count of 50,507,905 and mean read count of 52,054,095 (range 24,215,121–134,527,851). The *KneadData* pipeline (v0.10.0) was used to pre-process and decontaminate the sequencing reads. Raw reads were trimmed to a quality of Phred score 20, and adapters and reads below the minimum length were removed using the *trimmomatic* tool (Version 0.39) in the *kneadData* pipeline [16]. Quality reads were then mapped against the human reference genome database using *bowtie2* [17], and reads mapped to the human genome were removed from the data. Taxonomic profiling of the filtered sequence data was performed using *MetaPhlAn* (v3.0.0.0) [18], with the database mpa_v30_CHOCOPhlAn_201901. Functional read profiling was performed using *HUMAnN3* (v3.0.0) [18] including *MetaPhlAn*, *DIAMOND* 0.9.36 [19], and the databases uniref90 (v201901) [20] and mpa_v30_CHOCOPhlAn_201901, and we identified 502 pathways from the MetaCyc database (metacyc.org).

To identify gut microbial functional features (GMM and KEGG gut metabolic modules), we used our annotated functional genes data [21]. Regrouped and renamed gene family data from HUMAnN were grouped based on the KEGG module and GMMs using GMM.v1.07 from the GOMixer r-package (available at raeslab.org/gomixer) [22]. To test for differences in the distributions of KEGG modules and GMMs between HF and healthy controls, we used two-tailed Wilcoxon tests with Benjamini-Hochberg (BH) correction for multiple comparisons. Correlation analysis with Spearman’s correlation was performed to assess the concordance between microbial functional annotations (KEGG and GMM modules) from the HF study cohort and the MetaCardis cohort.

### Alpha and beta diversity analyses

Taxonomic profiles obtained from MetaPhlAn (v3.0.0.0) [18] were imported and analyzed using the *phyloseq* v1.40.0 and *Maaslin2* packages [23, 24] in R. To evaluate the taxonomic and functional richness, as well as diversity, we employed several indices including the Shannon index, inverse Simpson index, and Chao1 index. Beta-diversity was assessed by calculating the Bray–Curtis (BC) dissimilarity index. Ordinations were constructed using *Non-Metric Multidimensional Scaling (NMDS)* analysis. Multivariable *PERMANOVA*, with age, sex, and BMI as covariates, was performed using the *vegan::adonis2* function between HF groups limiting the 999 permutations using the BH method to control for multiple comparisons.

### Differential abundance analyses

To test for differences in the relative abundance differences between groups, we used *Multivariable Association Discovery in Population-scale Meta-omics Studies* (MaAsLin2) which is a modified linear model normalized for use in sparse, compositional microbial communities (https://huttenhower.sph.harvard.edu/maaslin) [24]. Age, sex, and BMI were included as covariates. Analyses were carried out at the genus and species level.

The microbial dysbiosis index [25] was calculated for each sample, based on differentially abundant bacterial taxa from the MaAsLin2 analysis on species level. The microbial dysbiosis index was calculated as log_10_ (sum of the abundances of the bacterial species increased in HF/sum of the abundances of species decreased in HF).

### Circulating markers

All participants had been fasting overnight prior to blood sampling. Serum and EDTA-plasma were separated by centrifugation at room temperature and 4 °C, respectively, within 1 h of collection. The blood samples were stored at – 80 °C. An electrochemiluminescence immunoassay (ECLIA) was used to detect levels of NT-ProBNP as part of routine clinical care (Roche Diagnostics, Mannheim, Germany).

ImP in serum was measured at BEVITAL (bevital.no) using a targeted metabolomics approach using a modification of a published liquid chromatography–tandem mass spectrometry (LC–MS/MS) method [26]: the method was complemented with ImP using non-labeled ImP (Sigma-Aldrich, St. Louis, MO 63178 USA) as calibrator and N1-methylnicotinamide-d4 (mNAM-d4) as labeled internal standard. The internal standard (purity > 95%) was obtained from PharmAgra Labs (40 McLean Road, Brevard, NC 28712). ImP was detected by positive-ion multiple reaction monitoring (MRM) at 141.1/123 m/z and mNAM-d4 at 141.3/78 m/z, and the retention times were 3.4 and 2.9 min, respectively. The lower limit of detection (LOD (S/N > 3) for ImP was 2 nmol/L. Within- and between-run coefficients of variation (CVs) were ≤ 5.4%.

Concentrations of C-reactive protein (CRP) was analyzed by ELISA (DRG Instruments, Marburg/Lahn, Germany). The inter-assay coefficients of variation for NT-ProBNP and CRP were 5% and 6.9% respectively. Commercially available enzyme-linked immunosorbent assays (ELISAs) were used to measure LPS-binding protein (LBP), intestinal fatty acid-binding protein (I-FABP; sourced from Hycult Biotech, Uden, the Netherlands), and soluble CD14 (sCD14) from R&D Systems Europe (Abingdon, Oxon, UK). The inter-assay coefficients of variation for LPS, LBP, I-FABP, and sCD14 were 2.8%, 9.6%, 14.4%, and 7.8%, respectively.

### Food frequency questionnaires

The HF patients completed a self-administrated, validated Norwegian food frequency questionnaire, aiming to reflect dietary habits over the past year. The questionnaire contained around 180 food items, with serving size alternatives specified in household units and calculated in grams using the software developed at the Institute for Nutrition Research, University of Oslo [27].

### Statistical analysis

For descriptive statistics, continuous variables were presented mean ± standard deviation. Categorical variables were presented as numbers and percent. Two-sided Wilcoxon tests were used to test for differences in distributions of continuous variables. Multivariable linear regression was used to estimate associations where age, sex, and BMI were included as covariates. ImP, CRP, LBP, sCD14, and IFABP were log (base 2)-transformed in regression analyses. The relationships between nutritional variables from the food questionnaires and serum ImP levels were tested using Spearman’s correlations. To identify the taxa that were most predictive of ImP levels, we used a random forest model with ImP residuals (adjusted for age, sex, BMI, and type 2 diabetes) as dependent variable and bacterial taxa as independent variables using the randomForest package. Internal validity was tested using 200 resamplings (bootstrapping) of the random forest model fitting procedure, and taxa were ranked based on how often they were selected among the top 20 most predictive taxa within each resampled dataset. Statistical analyses and data visualizations were done in R (v. 3.3.2).

## Results

### HF encompasses specific compositional and functional shifts in the gut microbiota

To characterize the gut microbiome profiles for composition and potential functional pathways associated with HF, we performed shotgun sequencing of the total fecal genomic DNA in a Norwegian cohort of subjects with heart failure (HF, *n* = 166) and healthy controls (HC, *n* = 69). Subjects with HF were on average older (60.2 vs 51.2 years) and had a higher BMI (28.3 vs 25.6 m^2^/kg) compared to the healthy controls (Table 1). All comparisons between subjects with HF and controls are therefore adjusted for age, BMI, and sex, as described in the “ Methods” section. A flow chart of the study design is shown in Fig. 1A.

**Table 1:** Demographic and clinical characteristics of study participants

**Characteristics**	**Healthy controls (HC, *n* = 69)**	**Heart failure (HF**, *n* = 166)	***p*-value**
Age (years)	51.7 (3.7)	60.2 (9.2)	< 0.001
Sex, female, *n* (%)	35 (51%)	44 (27%)	< 0.001
BMI, m^2^/kg	25.6 (3.3)	28.3 (5.1)	< 0.001
Smoke, *n* (%):			< 0.001
Ex-smoker	0 (0%)	25 (16%)	
Non-smoker	61 (88%)	82 (53%)	
Etiology of heart failure, *n* (%):			
Ischemic		80 (48%)	
Non-ischemic		76 (46%)	
NYHA class, *n* (%):			
II		106 (64%)	
III		59 (36%)	
LVEF, (%)		28.7 (7.1)	
Diabetes, *n* (%)		38 (23%)	
Hypertension, *n* (%)		46 (38%)	

Values are expressed as mean ± standard deviation, or percentage. *BMI* body mass index, *NYHA* New York Heart Association functional classification, *LVEF* left ventricular ejection fraction. Statistical significance was tested using Wilcoxon tests (age and BMI) or Fisher’s exact test (sex and smoking status)

The gut microbiota composition was altered in HF, supported by a statistically significant difference in multivariable Bray–Curtis dissimilarity indices (Fig. 1B). We used three distinct indices to assess functional gene richness and diversity in HF and healthy controls. The Shannon diversity index and Inverse Simpson index revealed statistically significantly reduced diversity and richness in HF subjects compared to HC (Fig. 1C, D). Disease status had a statistically significant effect on both indices also when adjusting for age, sex, and BMI (linear regressions *p* = 0.0044 and *p* = 0.027, respectively). HF-related dysbiosis appeared unrelated to both HF etiology and severity (Additional file 2: Fig. S1 A, B).

We next evaluated the taxonomic phyla driving the microbial compositional shifts. Subjects with HF displayed a significantly lower abundance of *Firmicutes* and *Actinobacteria*, and a higher relative abundance of *Bacteroidetes* compared to healthy controls (Fig. 1E and Additional file 3: Table S1-3), which was in line with previously published data on HF [3, 4, 11, 28]. At the genus level, we observed several differences between HF and healthy controls, including elevated abundance of genus *Hungatella*, *Oribacterium*, *Campylobacter*, *Coprobacillus*, *Intestinimonas*, *Lachnoclostridium*, and *Eisenbergiella* and reduced abundance of the genera *Actinomyces*, *Adlercreutzia*, *Bifidobacterium*, *Asaccharobacter*, *Gemmiger*, *Coprococcus*, *Faecalibacterium*, *Fusicatenibacter*, *Enterorhabdus*, *Olsenella*, *Dorea*, *Anaerostipes*, and *Roseburia* (Additional file 2: Fig. S1 C). In subjects with HF compared to HC, after adjusting for covariates (e.g. age, sex, and BMI) and multiple comparisons (*Q*_FDR_ < 0.05), four bacterial species (*Clostridium bolteae*, *Anaerotruncus colihominis*, *Clostridium symbiosum*, and *Streptococcus mutans*) had a higher abundance, whereas five bacterial species (*Faecalibacterium prausnitzii*, *Dorea formicigenerans*, *Actinomyces graevenitzii*, *Asaccharobacter celatus*, and *Adlercreutzia equolifaciens*) had a reduced abundance (Fig. 1E and Additional file 2: Fig. S2). These findings were consistent even when we performed a sub analysis in a subgroup of HF and HC matched for age, sex, and BMI (Additional file 2: Fig. S3 and Additional file 3: Table S4). The differentially abundant taxa in HF compared to HC were included to calculate the HF-related dysbiosis index, demonstrating the depletion of SCFA producing microbes on species level, including *F. prausnitzii*, a known producer of butyrate, and *D. formicigenerans*, a known producer of acetate and propionate [3, 5].

Next, we evaluated the shifts in potential microbial functions in HF. From the multivariable MaAsLin2 analysis on MetaCyc-based pathways, 52 pathways exhibited significant difference in abundance between HF and healthy controls. Most of these pathways were downregulated in HF (*Q*_FDR_ < 0.05, Additional file 2: Fig. S4 A). Employing the gut specific metabolic modules (GMMs), we identified 35 metagenome functions based on Kyoto Encyclopedia of Genes and Genomes (KEGG) and nine GMMs modules significantly altered in abundance in HF compared to healthy controls (*Q*_FDR_ < 0.05, Additional file 2: Fig. S4 B, C). Among these, four GMMs were highly enriched in HF, including metabolism of amino acids (MF0030_glutamate degradation I, MF0058_lysine degradation II and MF0052_arginine degradation II) and SCFA production (MF0093_propionate production I). To confirm our findings, we tested if the microbial functional shift in HF was consistent with deposited data from the MetaCardis consortium [2] which included subjects with HF and healthy controls. There was a modest, yet significant correlation between HF-related functional shifts between the two cohorts (*r* = 0.4, *p* < 2 × 2e^−16^) although this should be interpreted with caution as different methods have been applied (KEGGs and GMMs) (Additional file 2: Fig. S4 D).

### Imidazole propionate (ImP) is increased in HF and associated with HF-related dysbiosis

**Fig. 2:**
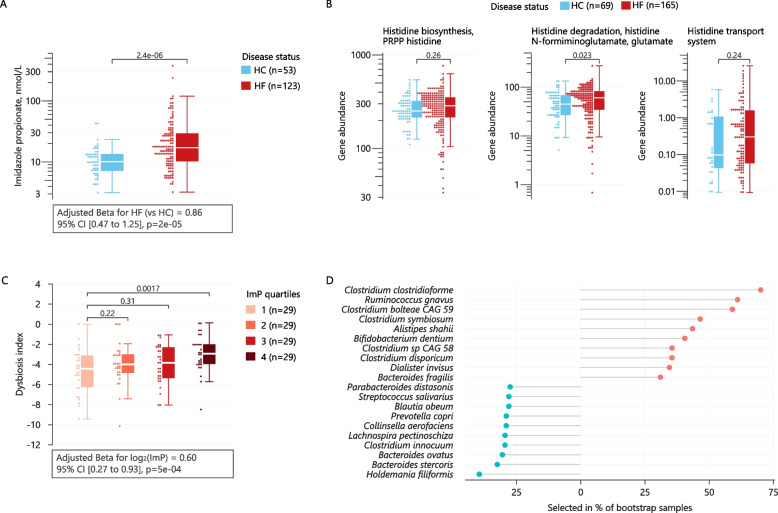
Imidazole propionate is associated with dysbiosis in heart failure. **A** Serum levels of ImP in heart failure (HF) and healthy controls (HC) is shown as box plots and dot plots. Statistical significance was tested using Wilcoxon tests. A multivariable linear regression model was fitted to estimate whether the difference remained after adjusting for age and sex (model estimates are shown below the plot). **B** Distributions of gene abundances based on Kyoto Encyclopedia of Genes and Genomes (KEGGs) for histidine biosynthesis, degradation, and transport in HC and HF are shown as box plots and dot plots. Statistical significance was tested using Wilcoxon tests. **C** Distributions of the dysbiosis index (material and methods) are shown for quartiles of serum ImP. Statistical significance was calculated using a Wilcoxon test and a linear regression analysis where ImP was modeled continuously (log_2_-transformed) with age, sex, and BMI as covariates (model estimates for log_2_(ImP) is shown below the plot). **D** Top bacterial species associated with serum ImP from a bootstrapped random forest analysis. The bacterial species’ ranks are indicated by how often they were selected in the bootstrap repetitions (material and methods). Red dots: elevated in HF; blue dots: decreased in HF. See also Additional file 2 Table S5

As some of the species exhibiting the largest differences in abundance between HF and healthy controls, such as *C. bolteae*, *C. symbiosum*, and *F. prausnitzii*, have been linked to production of ImP [12], a microbial histidine metabolite recently found elevated in HF [13], we next measured circulating levels of this metabolite in the current cohort. Subjects with HF displayed significantly higher levels of ImP compared to healthy controls (Fig. 2A), and the difference persisted when adjusting for known confounding factors (age, sex and BMI, linear regression *p* < 0.001).

Upon evaluating bacterial functions related to histidine metabolism, we observed a significant increase in bacterial KEGG orthologs involved in histidine degradation and transport compared to healthy controls (Fig. 2B). We therefore sought to investigate whether the disease-related microbiota alterations in HF could contribute to circulating ImP levels. To this end, we investigated whether ImP associated with the HF-related dysbiosis index. Indeed, elevated ImP levels associated with a higher degree of dysbiosis, an effect that persisted after adjusting for age, sex, and BMI (Fig. 2C). Using random forest with resampling, we observed specific bacterial species associated with ImP production in HF with high internal validity (Fig. 2D). Several of the bacterial species associated with ImP production were also differentially abundant in HF relative to healthy controls (Fig. 1E). Of note, a group of *Clostridia* (*clostridioforme*, *bolteae*, and *symbiosum*) and *Ruminococcus gnavus* were the most important bacterial species positively associated with ImP after adjustment for age, sex, BMI, and diabetes status (Additional file 3: Table S5). These bacteria have previously been found to be more abundant in individuals with type 2 diabetes and prediabetes [29, 30], and in inflammatory bowel disease [31], and are putative ImP-producers [12, 32]. Furthermore, bacteria with anti-inflammatory properties such as *Bacteroides ovatus* [33, 34] and *Prevotella copri* [35] were negatively correlated with ImP levels. Thus, these observations suggest a potential link between a pro-inflammatory microbiota composition and ImP production and are in line with the initial observation that ImP levels were elevated in individuals with gut inflammation [36].

### ImP is associated with markers of intestinal permeability and systemic inflammation

**Fig. 3:**
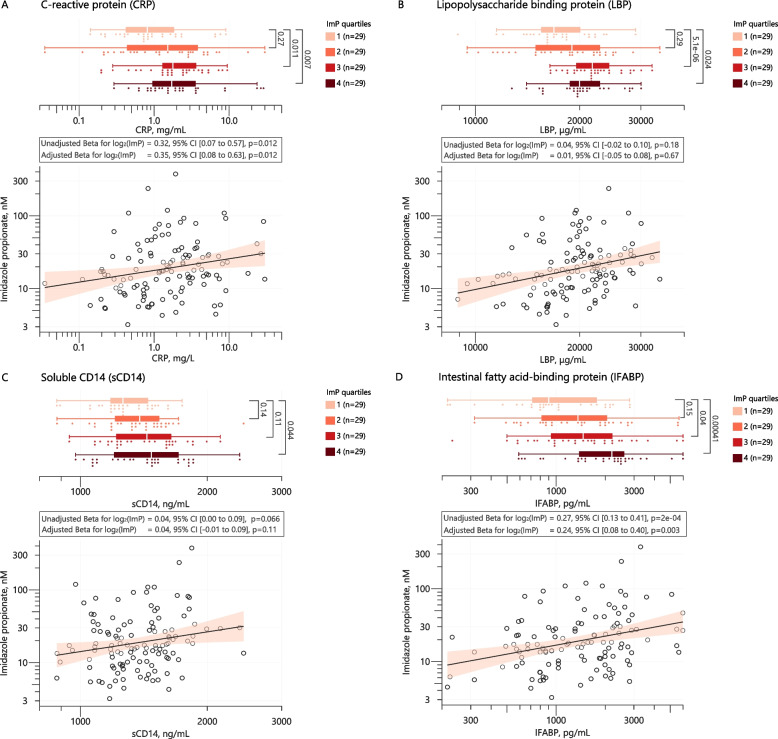
Imidazole propionate is associated with increased systemic inflammation and gut permeability markers. Distributions of CRP (**A**), LBP (**B**), sCD14 (**C**), and I-FABP (**D**) are shown as box plots and dot plots. Statistical significance was tested using Wilcoxon tests. Below each plot, model estimates from univariable and multivariable (age, sex, and BMI) linear regressions and scatterplots of ImP versus each of the four markers are shown. CRP, LBP, sCD14, and IFABP were log_2_-transformed in the regression analyses. CRP, C-reactive protein; I-FABP, intestinal fatty acid binding protein; LBP, LPS-binding protein; sCD14, soluble CD14

Several studies have shown that microbiota-driven low-grade inflammation is detrimental for cardiometabolic diseases [37], with intestinal permeability and microbial by-products including LPS as potential contributing factors. For this reason, we evaluated if ImP serum levels were associated with low-grade inflammation and markers of intestinal permeability in subjects with HF. Subjects with ImP levels above the 75th percentile had higher levels of C-reactive protein (CRP) and LPS-driven inflammatory response proteins (LPS binding protein, LBP and soluble CD14, sCD14) compared to subjects below the 25th percentile (*p* = 0.007, *p* = 0.024, and *p* = 0.044, respectively; Fig. 3A–C). Moreover, serum levels of intestinal fatty-acid binding protein (I-FABP), a marker of intestinal permeability, were also significantly higher in subjects with high levels of ImP (*p* < 0.001, Fig. 3D). When modeling ImP as a continuous variable, ImP was only positively associated with CRP and IFABP, effects that remained significant when adjusting for age, sex, and BMI. Taken together, our data suggest that ImP is associated with increased systemic inflammation which could potentially be driven by increased intestinal permeability.

### Circulating ImP levels are associated with severity and etiology of HF

**Fig. 4:**
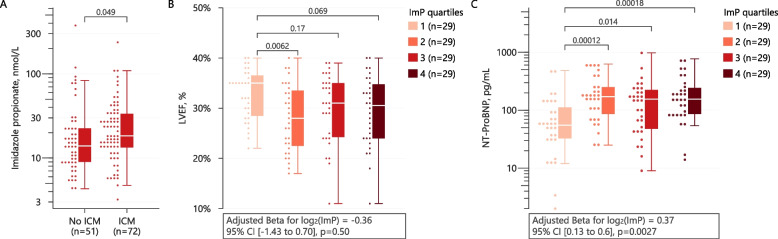
Imidazole propionate in relation to severity and etiology of heart failure. Imidazole propionate serum levels (quartiles) are shown in relation to etiology of heart failure (**A**), percentage of LVEF (**B**), and serum levels of NT-ProBNP (**C**) as box plots and dot plots. Statistical significance was tested using Wilcoxon tests. For LVEF and NT-ProBNP, univariable and multivariable (age, sex, and BMI) linear regression model estimates for ImP are shown. LVEF, left ventricular ejection fraction; NT-proBNP, amino-terminal pro B-type natriuretic peptide

Next, we investigated whether HF-related dysbiosis or circulating ImP levels were related to etiology and severity of HF. First, subjects with ischemic HF displayed significantly higher levels of serum ImP compared to subjects with non-ischemic HF (Fig. 4A). Second, we observed significantly higher levels of ImP in individuals with impaired LV systolic function as measured by LVEF (Fig. 4B), but this effect was largely explained by age, sex, and BMI. Meanwhile, NT-proBNP, a marker of cardiac wall stress, was significantly increased with increasing ImP quartiles (Fig. 4C), and this was supported by a multivariable linear regression model with age, sex, and BMI included as covariates (*p* = 0.003). Finally, as previously shown in a non-HF population, subjects with T2D compared to subjects without displayed significantly higher levels of ImP (Additional file 2: Fig. S5) [12].

Taken together our data confirm and extend our recent findings [13] that ImP not only is increased in subjects with HF but also correlates with disease severity.

### Reduced fiber intake is associated with serum levels of imidazole propionate

An unhealthy dietary pattern has been shown to be a contributing determinant of circulating ImP in T2D [12], prompting us to also investigate whether this applied to the present HF cohort. While nutrient intake overall showed no clear associations with ImP, there was a weak but significant negative correlation between ImP and dietary fiber (Spearman’s rho =  − 0.2, *p* < 0.05), driven by a negative correlation between ImP and vegetables (Spearman’s rho =  − 0.24, *p* < 0.05, Additional file 2: Fig. S6).

## Discussion

The gut microbiota produces a large number of metabolites with potential impact on cardiometabolic diseases, but a clear link between the specific gut microbiota alterations associated with the disease and the circulating microbiota-derived metabolites has been lacking [6]. Here, we show in the largest study of gut metagenomes in HF to date that some of the most enriched bacterial species also are strongly associated with higher circulating levels of the microbially derived metabolite ImP. Although the compositional shifts in the gut microbiota of subjects with HF compared with healthy controls are in line with other studies [2–5, 13, 38], the dysbiosis did not correlate with severity and etiology of HF, in contrast to circulating levels of the microbial-derived metabolite ImP.

Our results independently confirm and expand our recent observation that ImP levels are elevated in subjects with HF [13]. The gut microbial composition has been shown to differ within cities, regions, and countries [39, 40], whereas our previous study showed a consistent association between ImP and HF across geographically distinct cohorts [13]. Hence, metabolites such as ImP, rather than metagenomic data, can indeed serve as relevant indicators of altered microbial function across populations, potentially providing valuable insights into the metabolic activities of the microbiome.

In a search for bacterial species associated with ImP levels, we identified a group of *Clostridia* (*clostridioforme*, *bolteae*, and *symbiosum*), in addition to *R. gnavus*, *Erysipelatoclostridium ramosum*, and *Intestinibacter bartlettii*, all of which have previously been shown in type 2 diabetes to positively correlate with ImP production [12]. Accordingly, we found significantly higher ImP levels in subjects with HF and with T2D, and higher levels in subjects with ischemic cardiomyopathy, hence connecting this metabolite to important etiologies of chronic HF. So far, no studies have reported ImP in relation to coronary artery disease, but considering our findings, such studies are warranted.

Cardiometabolic diseases such as HF, atherosclerotic heart disease, and T2D are all partly driven by chronic low-grade inflammation, which could be a common mechanistic link between ImP and these conditions [1, 12]. ImP was originally identified as elevated in urine in subjects with gastrointestinal inflammation in the 1970s and has also been associated with inflammatory bowel disease [36, 41]. ImP has been shown to be associated with pro-inflammatory cytokines in subject with type 2 diabetes, and in the present study, we found ImP levels to associate with higher levels of CRP [12] as well as sCD14, both reliable markers of systemic inflammation and monocyte activation, which are involved in HF pathogenesis [42].

A common finding of previous gut microbiota studies in cardiovascular disease has been depletion of bacteria with the potential of producing SCFAs. In this cohort, we found a depletion of SCFA-producing microbes on the species level, including *F. prausnitzii* and *D. formicigenerans* [43]. On a functional level, we identified pathways related to metabolism of amino acids and SCFA production, consistent with deposited data from the MetaCardis consortium [2]. SCFAs are the main energy source for colonocytes and therefore vital for maintaining the integrity of the mucosal barrier [11, 44]. We therefore hypothesized that the association between ImP and low-grade inflammation could be partly explained by an impaired gut mucosal barrier. Interestingly, ImP levels were associated with I-FABP, a marker of enterocyte damage, as well as LBP and sCD14, markers of LPS-driven inflammatory response.

A likely hypothesis is that ImP could contribute to a chronic low-grade inflammation observed in metabolic diseases [1]. ImP induces insulin resistance in vivo by inducing mTORC1 signaling through p38γ/δ, and MKK6-deficient mice lacking p38γ/δ do not develop heart hypertrophy [45, 46]. In addition, ImP inhibits AMPK signaling, which is a key sensor of cellular energy [47]. Since both pathways have been linked with cardiac fibrosis, hypertrophy, and HF [48, 49], ImP may contribute to HF through these mechanisms. However, further studies using chronic delivery of ImP in animal models of HF are required to clarify if and how ImP directly affects HF pathogenesis. It should also be noted that *Ruminococcus gnavus* has been associated with several chronic inflammatory diseases, possibly through its ability to produce proinflammatory polysaccharides [32, 50], which could also contribute to the association between ImP and gut inflammation.

Diet has major impact on gut microbiota composition and function, and our unadjusted analyses showed that ImP production was negatively associated with dietary intake of fiber and vegetables. It is known that long-term low-fiber diet can lead to a dysbiotic microbial environment. Here, we confirm this observation, as has been shown for type 2 diabetes, that also in HF an unhealthy diet with reduced intake of fiber and vegetables can induce changes in the microbiota with increased capacity to produce ImP [12, 51]. This fits with our previous observation that much of the HF-related dysbiosis, including depletion of SCFA producing microbes, is associated with low fiber intake [28].

The present study has some limitations including lack of an independent validation cohort to confirm the associations with changes in the gut microbiome, and our cohort did not include subjects with HF in NYHA class I or IV. Nevertheless, this is the largest study of gut metagenomes in HF to date, using a stringent correction for multiple comparisons, and together with our recent report of elevated ImP levels in HF [13], this makes a strong case for the involvement of ImP in cardiometabolic diseases including HF. Moreover, further studies are needed in order to understand the bacterial enzymes responsible for the ImP production beside the known pathways [52].

## Conclusions

In summary, our findings establish a link between disease-specific changes in the gut microbiota, the presence, etiology, and severity of HF, and the gut microbially produced metabolite ImP. Using an independent cohort, we extend on our recent data linking ImP to HF [13]. Intriguingly, our findings suggest that exploring strategies to inhibit bacterial ImP productions by directly targeting gut microbes (“drugging the bug”) may be a promising treatment strategy for HF and other cardiometabolic diseases in individuals with elevated ImP levels.

### Supplementary Information

**Additional file 1:** **Additional file 1. **The document describes the primary/secondary outcomes of the GutHeart clincal trial.

**Additional file 2:** **Fig. S1** **A**. box- and dotplots showing the distributions of Shannon diversity index, inverse Simpson and Chao1 in subjects with heart failure according to etiology. **B**. Correlation matrix plot for Shannon diversity index, inverse Simpson and Chao1 and LVEF, NT-Pro-BNP. **C**. A multivariable MaAsLin2 analysis shows which bacterial genera were elevated and decreased in heart failure after adjustment for age, sex and BMI. **Fig. S2**. Distributions of differentially abundant bacterial species in heart failure (HF) compared to healthy controls (HC) after adjustment for age, sex and BMI. **Fig. S3**. Distributions of differentially abundant bacterial species in heart failure (HF) compared to healthy controls (HC), in a subsample with comparable age (± 1 year) and BMI (± 1 kg/m2). **Fig. S4.** A. A multivariable MaAsLin2 analysis shows which bacterial pathways were elevated and decreased in heart failure after adjustment for age, sex and BMI. **B, C**. Differentially expressed bacterial functions in HF vs HC according to KEGG and GMM gut metabolic modules. **D**. Scatterplot of pooled KEGG and GMM bacterial functions from the current study and the MetaCardis study. **Fig. S5**. Box- and dotplots showing the distributions of imidazole propionate serum levels in HC and HF patients with or without type 2 diabetes (T2D). **Fig. S6**. A Correlation matrix plot for imidazole propionate serum levels and macronutrients and food categories in subjects with heart failure.

**Additional file 3:** **Table S1**. Distribution and abundance of taxa in healthy controls and heart failure subjects on Phylum level. **Table S2**. Distribution and abundance of taxa in Healthy controls and heart failure subjects on Genus level. **Table S3**. Demographic and clinical characteristics of subsample study participants, matched for age, gender, BMI. **Table S4**. Distribution and abundance of taxa in Healthy controls and heart failure subjects on Species level. **Table S5**. mOTUs correlated with ImP residuals. **Table S6**. Description of the gut microbial functional variation using KEGG and GMM modules.

## Data Availability

For reasons related to Norwegian legislation and the participant consent forms, the data from the metagenomic sequencing are not available in public repositories. The data are however available upon reasonable request to Prof. Marius Trøseid, following the establishment of a material and data transfer agreement between the institutions and the approval of an amendment application to the Regional Committee for Medical and Health Research Ethics to ensure that the aim of the planned research is covered by the participant consent forms. All the code necessary to replicate the key findings presented in this manuscript is accessible at https://github.com/sajanraju/HF_and_IMP_GMED.

## References

[1] Tang WHW, Bäckhed F, Landmesser U, Hazen SL (2019). Intestinal microbiota in cardiovascular health and disease. J Am Coll Cardiol.

[2] Fromentin S, Forslund SK, Chechi K, Aron-Wisnewsky J, Chakaroun R, Nielsen T (2022). Microbiome and metabolome features of the cardiometabolic disease spectrum. Nat Med.

[3] Cui X, Ye L, Li J, Jin L, Wang W, Li S (2018). Metagenomic and metabolomic analyses unveil dysbiosis of gut microbiota in chronic heart failure patients. Sci Rep.

[4] Kummen M, Mayerhofer CCK, Vestad B, Broch K, Awoyemi A, Storm-Larsen C (2018). Gut microbiota signature in heart failure defined from profiling of 2 independent cohorts. J Am Coll Cardiol.

[5] Kamo T, Akazawa H, Suda W, Saga-Kamo A, Shimizu Y, Yagi H (2017). Dysbiosis and compositional alterations with aging in the gut microbiota of patients with heart failure. PLoS ONE.

[6] Krautkramer KA, Fan J, Bäckhed F (2021). Gut microbial metabolites as multi-kingdom intermediates. Nat Rev Microbiol.

[7] Trøseid M, Ueland T, Hov JR, Svardal A, Gregersen I, Dahl CP (2015). Microbiota-dependent metabolite trimethylamine-N-oxide is associated with disease severity and survival of patients with chronic heart failure. J Intern Med.

[8] Tang WHW, Wang Z, Fan Y, Levison B, Hazen JE, Donahue LM (2014). Prognostic value of elevated levels of intestinal microbe-generated metabolite trimethylamine-N-oxide in patients with heart failure: refining the gut hypothesis. J Am Coll Cardiol.

[9] Mayerhofer CCK, Ueland T, Broch K, Vincent RP, Cross GF, Dahl CP (2017). Increased secondary/primary bile acid ratio in chronic heart failure. J Card Fail.

[10] Romano KA, Nemet I, Prasad Saha P, Haghikia A, Li XS, Mohan ML (2023). Gut microbiota-generated phenylacetylglutamine and heart failure. Circ Heart Fail.

[11] Trøseid M, Andersen GØ, Broch K, Hov JR (2020). The gut microbiome in coronary artery disease and heart failure: Current knowledge and future directions. EBioMedicine.

[12] Molinaro A, Bel Lassen P, Henricsson M, Wu H, Adriouch S, Belda E (2020). Imidazole propionate is increased in diabetes and associated with dietary patterns and altered microbial ecology. Nat Commun.

[13] Molinaro A, Nemet I, Bel Lassen P, Chakaroun R, Nielsen T, Aron-Wisnewsky J (2023). Microbially Produced Imidazole Propionate Is Associated With Heart Failure and Mortality. JACC Heart Fail..

[14] Awoyemi A, Mayerhofer C, Felix AS, Hov JR, Moscavitch SD, Lappegård KT (2021). Rifaximin or Saccharomyces boulardii in heart failure with reduced ejection fraction: Results from the randomized GutHeart trial. EBioMedicine.

[15] Costea PI, Zeller G, Sunagawa S, Pelletier E, Alberti A, Levenez F (2017). Towards standards for human fecal sample processing in metagenomic studies. Nat Biotechnol.

[16] Bolger AM, Lohse M, Usadel B (2014). Trimmomatic: a flexible trimmer for Illumina sequence data. Bioinformatics.

[17] Langmead B, Salzberg SL (2012). Fast gapped-read alignment with Bowtie 2. Nat Methods.

[18] Beghini F, McIver LJ, Blanco-Míguez A, Dubois L, Asnicar F, Maharjan S, et al. Integrating taxonomic, functional, and strain-level profiling of diverse microbial communities with bioBakery 3. Elife. 2021;10:e65088 10.7554/eLife.65088 PMC8096432 33944776

[19] Buchfink B, Xie C, Huson DH (2015). Fast and sensitive protein alignment using DIAMOND. Nat Methods.

[20] Suzek BE, Huang H, McGarvey P, Mazumder R, Wu CH (2007). UniRef: comprehensive and non-redundant UniProt reference clusters. Bioinformatics.

[21] Vieira-Silva S, Falony G, Darzi Y, Lima-Mendez G, Garcia Yunta R, Okuda S (2016). Species–function relationships shape ecological properties of the human gut microbiome. Nat Microbiol.

[22] Darzi Y, Falony G, Vieira-Silva S, Raes J (2016). Towards biome-specific analysis of meta-omics data. ISME J.

[23] McMurdie PJ, Holmes S (2013). phyloseq: an R package for reproducible interactive analysis and graphics of microbiome census data. PLoS One.

[24] Mallick H, Rahnavard A, McIver LJ, Ma S, Zhang Y, Nguyen LH (2021). Multivariable association discovery in population-scale meta-omics studies. PLOS Comput Biol..

[25] Gevers D, Kugathasan S, Denson LA, Vázquez-Baeza Y, Van Treuren W, Ren B (2014). The treatment-naive microbiome in new-onset Crohn’s disease. Cell Host Microbe.

[26] Midttun Ø, Hustad S, Ueland PM (2009). Quantitative profiling of biomarkers related to B-vitamin status, tryptophan metabolism and inflammation in human plasma by liquid chromatography/tandem mass spectrometry. Rapid Commun Mass Spectrom.

[27] Andersen LF, Solvoll K, Johansson LR, Salminen I, Aro A, Drevon CA (1999). Evaluation of a food frequency questionnaire with weighed records, fatty acids, and alpha-tocopherol in adipose tissue and serum. Am J Epidemiol.

[28] Mayerhofer CCKK, Kummen M, Holm K, Broch K, Awoyemi A, Vestad B (2020). Low fibre intake is associated with gut microbiota alterations in chronic heart failure. ESC Hear Fail.

[29] Qin J, Li Y, Cai Z, Li S, Zhu J, Zhang F (2012). A metagenome-wide association study of gut microbiota in type 2 diabetes. Nature.

[30] Allin KH, Tremaroli V, Caesar R, Jensen BAH, Damgaard MTF, Bahl MI (2018). Aberrant intestinal microbiota in individuals with prediabetes. Diabetologia.

[31] Qiu P, Ishimoto T, Fu L, Zhang J, Zhang Z, Liu Y. The Gut Microbiota in Inflammatory Bowel Disease. Front Cell Infect Microbiol. 2022;12:733992. 10.3389/fcimb.2022.733992 PMC8902753 35273921

[32] Hall AB, Yassour M, Sauk J, Garner A, Jiang X, Arthur T (2017). A novel Ruminococcus gnavus clade enriched in inflammatory bowel disease patients. Genome Med.

[33] Ihekweazu FD, Fofanova TY, Queliza K, Nagy-Szakal D, Stewart CJ, Engevik MA (2019). Bacteroides ovatus ATCC 8483 monotherapy is superior to traditional fecal transplant and multi-strain bacteriotherapy in a murine colitis model. Gut Microbes.

[34] Fultz R, Ticer T, Ihekweazu FD, Horvath TD, Haidacher SJ, Hoch KM (2021). Unraveling the metabolic requirements of the gut commensal Bacteroides ovatus. Front Microbiol.

[35] Sandberg J, Kovatcheva-Datchary P, Björck I, Bäckhed F, Nilsson A (2019). Abundance of gut Prevotella at baseline and metabolic response to barley prebiotics. Eur J Nutr.

[36] van der Heiden C, Wadman SKK, De Bree PKK, Wauters EAKA (1972). Increased urinary imidazolepropionic acid, N-acetylhistamine and other imidazole compounds in patients with intestinal disorders. Clin Chim Acta.

[37] Valles-Colomer M, Menni C, Berry SE, Valdes AM, Spector TD, Segata N (2023). Cardiometabolic health, diet and the gut microbiome: a meta-omics perspective. Nat Med.

[38] Mayerhofer CCK, Awoyemi AO, Moscavitch SD, Lappegård KT, Hov JR, Aukrust P (2018). Design of the GutHeart—targeting gut microbiota to treat heart failure—trial: a phase II, randomized clinical trial. ESC Hear Fail.

[39] Deschasaux M, Bouter KE, Prodan A, Levin E, Groen AK, Herrema H (2018). Depicting the composition of gut microbiota in a population with varied ethnic origins but shared geography. Nat Med.

[40] He Y, Wu W, Zheng H-M, Li P, McDonald D, Sheng H-F (2018). Regional variation limits applications of healthy gut microbiome reference ranges and disease models. Nat Med.

[41] Vich Vila A, Hu S, Andreu-Sánchez S, Collij V, Jansen BH, Augustijn HE (2023). Faecal metabolome and its determinants in inflammatory bowel disease. Gut..

[42] Adamo L, Rocha-Resende C, Prabhu SD, Mann DL (2020). Reappraising the role of inflammation in heart failure. Nat Rev Cardiol.

[43] Morrison DJ, Preston T (2016). Formation of short chain fatty acids by the gut microbiota and their impact on human metabolism. Gut Microbes.

[44] Roediger WE (1982). Utilization of nutrients by isolated epithelial cells of the rat colon. Gastroenterology.

[45] Romero-Becerra R, Mora A, Manieri E, Nikolic I, Santamans AM, Montalvo-Romeral V, et al. MKK6 deficiency promotes cardiac dysfunction through MKK3-p38γ/δ-mTOR hyperactivation. Elife. 2022;11:e75250. 10.7554/eLife.75250 PMC9381040 35971771

[46] González-Terán B, López JA, Rodríguez E, Leiva L, Martínez-Martínez S, Bernal JA (2016). p38γ and δ promote heart hypertrophy by targeting the mTOR-inhibitory protein DEPTOR for degradation. Nat Commun.

[47] Koh A, Mannerås-Holm L, Yunn N-O, Nilsson PM, Ryu SH, Molinaro A (2020). Microbial Imidazole propionate affects responses to metformin through p38γ-dependent inhibitory AMPK phosphorylation. Cell Metab.

[48] Li Y, Li Z, Zhang C, Li P, Wu Y, Wang C (2017). Cardiac fibroblast–specific activating transcription factor 3 protects against heart failure by suppressing MAP2K3-p38 signaling. Circulation.

[49] Bairwa SC, Parajuli N, Dyck JRB (2016). The role of AMPK in cardiomyocyte health and survival. Biochim Biophys Acta - Mol Basis Dis.

[50] Henke MT, Brown EM, Cassilly CD, Vlamakis H, Xavier RJ, Clardy J. Capsular polysaccharide correlates with immune response to the human gut microbe Ruminococcus gnavus. Proc Natl Acad Sci. 2021;118:e2007595118. 10.1073/pnas.2007595118 PMC8157926 33972416

[51] Wu GD, Chen J, Hoffmann C, Bittinger K, Chen Y-Y, Keilbaugh SA (2011). Linking long-term dietary patterns with gut microbial enterotypes. Science.

[52] Koh A, Molinaro A, Ståhlman M, Khan MT, Schmidt C, Mannerås-Holm L (2018). Microbially produced imidazole propionate impairs insulin signaling through mTORC1. Cell.

