# Supplementary material for: Microbial-derived imidazole propionate links the heart failure-associated microbiome alterations to disease severity
Source: Genome Med. 2024 Feb 8;16:27. doi: 10.1186/s13073-024-01296-6 (PMC10854170; doi:10.1186/s13073-024-01296-6)
Supplement: Supplementary file 1 — Additional file 1: Additional file 1. The document describes the primary/secondary outcomes of the GutHeart clincal trial. [file 13073_2024_1296_MOESM1_ESM.docx]

**Additional file 1**

The primary end point of the GutHeart study was changes in left ventricular ejection fraction after 3 months of intervention, whereas secondary end points were changes from baseline to 3 months in Chao 1 diversity index, circulating trimethylamine-N-oxide, C-reactive protein and occurrence of adverse events, and the main result has been previously published [1]. The results of the present study are not among the primary or secondary end points of the GutHeart trial. The study protocol has been published separately [2].

Secondary endpoints according to protocol were:

- Gut microbiota composition analyzed by sequencing of 16s ribosomal RNA gene (Illumina chemistry), including between group differences (beta diversity) and time-dependent changes in individuals (alpha diversity), as well as the abundance of individual taxa.
- Microbiota-related circulating metabolites including TMAO, plasma lipidomics including primary and secondary bile acids, plasma metabolomics.
- Other measures of cardiac function: left ventricular end systolic and end diastolic volumes (LVESV, LVEDV), regional wall motion score index (WMSI) as measured by echocardiography, NYHA class, and neurohormones.
- NT-proBNP.
- Inflammatory, anti-inflammatory and gut leakage markers in plasma, serum, peripheral blood, PBMC: i.e. CRP as a reliable marker of up-stream inflammation, plasma levels of LPS as a marker of gut leakage, sCD163 and neopterin as markers of innate immunity, sCD25 as a marker of T cell activation, von Willebrand factor as a marker of endothelial cell activation, sCD40L as a marker of platelet-related inflammation, IL-10 as an anti-inflammatory cytokine, and TNF, IL-1, IL-7 and IL-18 as pro-inflammatory cytokines.
- Health-related quality of life score as measured by the Minnesota Living with Heart Failure Questionnaire
- Functional capacity (6 minutes walk test).
- Endothelial function assessed by Endo-PAT® (PAT=peripheral arterial tone)
- LVEF, 6 minutes walk test and blood samples at 6 months.
- Safety, including side effects and withdrawal.

**References**

1. Awoyemi A, Mayerhofer C, Felix AS, Hov JR, Moscavitch SD, Lappegård KT, et al. Rifaximin or Saccharomyces boulardii in heart failure with reduced ejection fraction: Results from the randomized GutHeart trial. EBioMedicine. 2021;70:103511.

2. Mayerhofer CCK, Awoyemi AO, Moscavitch SD, Lappegård KT, Hov JR, Aukrust P, et al. Design of the GutHeart—targeting gut microbiota to treat heart failure—trial: a Phase II, randomized clinical trial. ESC Hear Fail. 2018;5:977–84.
