# Supplementary material for: Microbial-derived imidazole propionate links the heart failure-associated microbiome alterations to disease severity
Source: Genome Med. 2024 Feb 8;16:27. doi: 10.1186/s13073-024-01296-6 (PMC10854170; doi:10.1186/s13073-024-01296-6)
Supplement: Supplementary file 2 — Additional file 2: Fig. S1 A. box- and dotplots showing the distributions of Shannon diversity index, inverse Simpson and Chao1 in subjects with heart failure according to etiology. B. Correlation matrix plot for Shannon diversity index, inverse Simpson and Chao1 and LVEF, NT-Pro-BNP. C. A multivariable MaAsLin2 analysis shows which bacterial genera were elevated and decreased in heart failure after adjustment for age, sex and BMI. Fig. S2. Distributions of differentially abundant bacterial species in heart failure (HF) compared to healthy controls (HC) after adjustment for age, sex and BMI. Fig. S3. Distributions of differentially abundant bacterial species in heart failure (HF) compared to healthy controls (HC), in a subsample with comparable age (± 1 year) and BMI (± 1 kg/m2). Fig. S4. A. A multivariable MaAsLin2 analysis shows which bacterial pathways were elevated and decreased in heart failure after adjustment for age, sex and BMI. B, C. Differentially expressed bacterial functions in HF vs HC according to KEGG and GMM gut metabolic modules. D. Scatterplot of pooled KEGG and GMM bacterial functions from the current study and the MetaCardis study. Fig. S5. Box- and dotplots showing the distributions of imidazole propionate serum levels in HC and HF patients with or without type 2 diabetes (T2D). Fig. S6. A Correlation matrix plot for imidazole propionate serum levels and macronutrients and food categories in subjects with heart failure. [file 13073_2024_1296_MOESM2_ESM.docx]

**
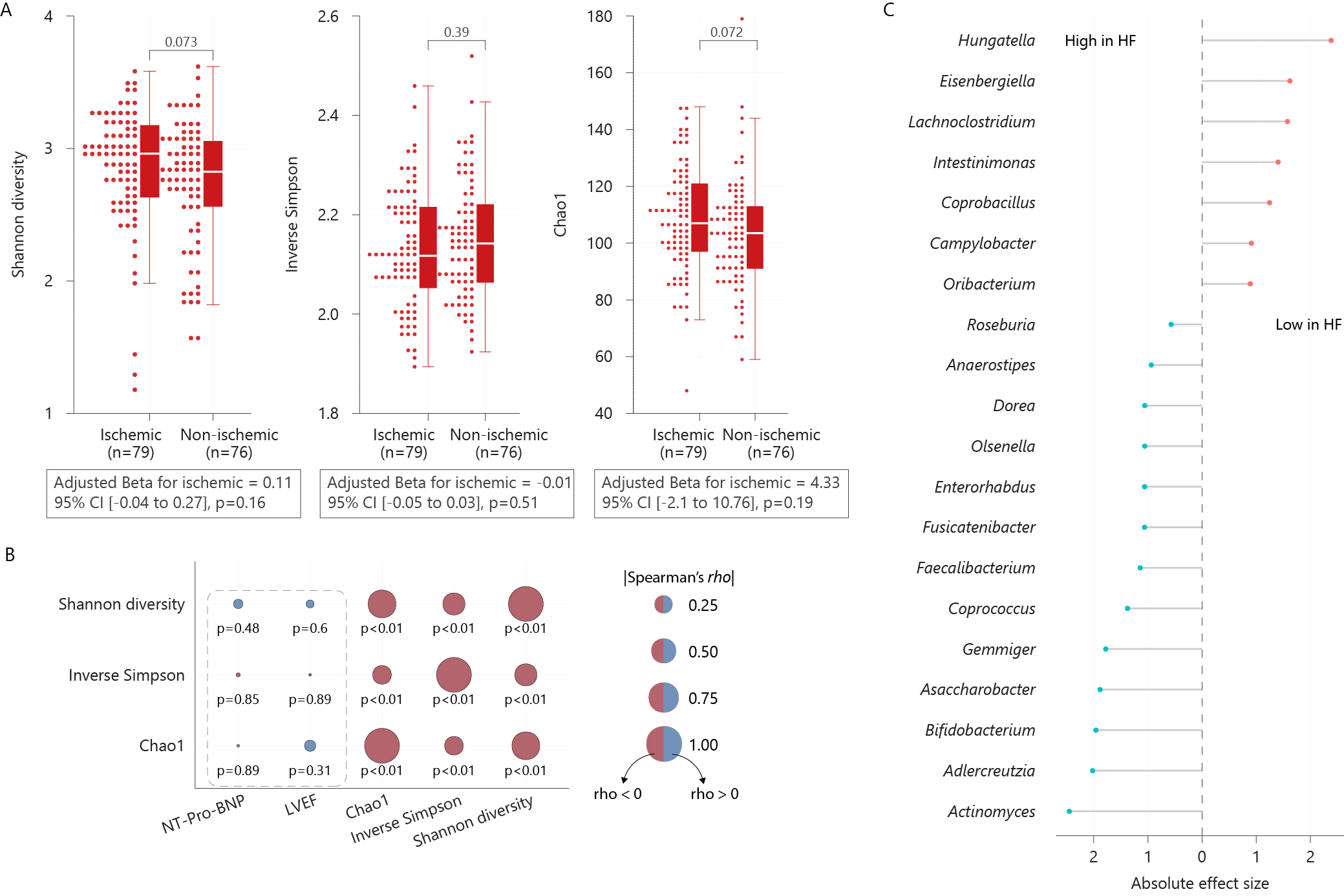
**

**Fig. S1. Dysbiosis in heart failure does not correlate with etiology or severity. A**. Distributions of Shannon diversity index, inverse Simpson and Chao1 in subjects with heart failure according to etiology is shown as box- and dotplots. Statistical significance was tested using Wilcoxon tests. Multivariable (age, sex, and BMI) linear regression model estimates for ischemic (versus non-ischemic) are shown below each plot. **B.** Correlation matrix plot for Shannon diversity index, inverse Simpson and Chao1 and LVEF, NT-Pro-BNP is shown. Statistical significance of the bivariate correlations was tested using Spearman’s correlation (p-values indented in the plot). The size and colors of the bubbles indicate the effect sizes and directions of the correlations. **C.** A multivariable MaAsLin2 analysis of heart failure versus healthy controls was performed to estimate which bacterial genera were elevated and decreased in heart failure after adjustment for age, sex and BMI. The dots indicate the absolute effect sizes, i.e. the degree to which the individual genera were different in heart failure and healthy controls. ICM: Ischemic cardiomyopathy


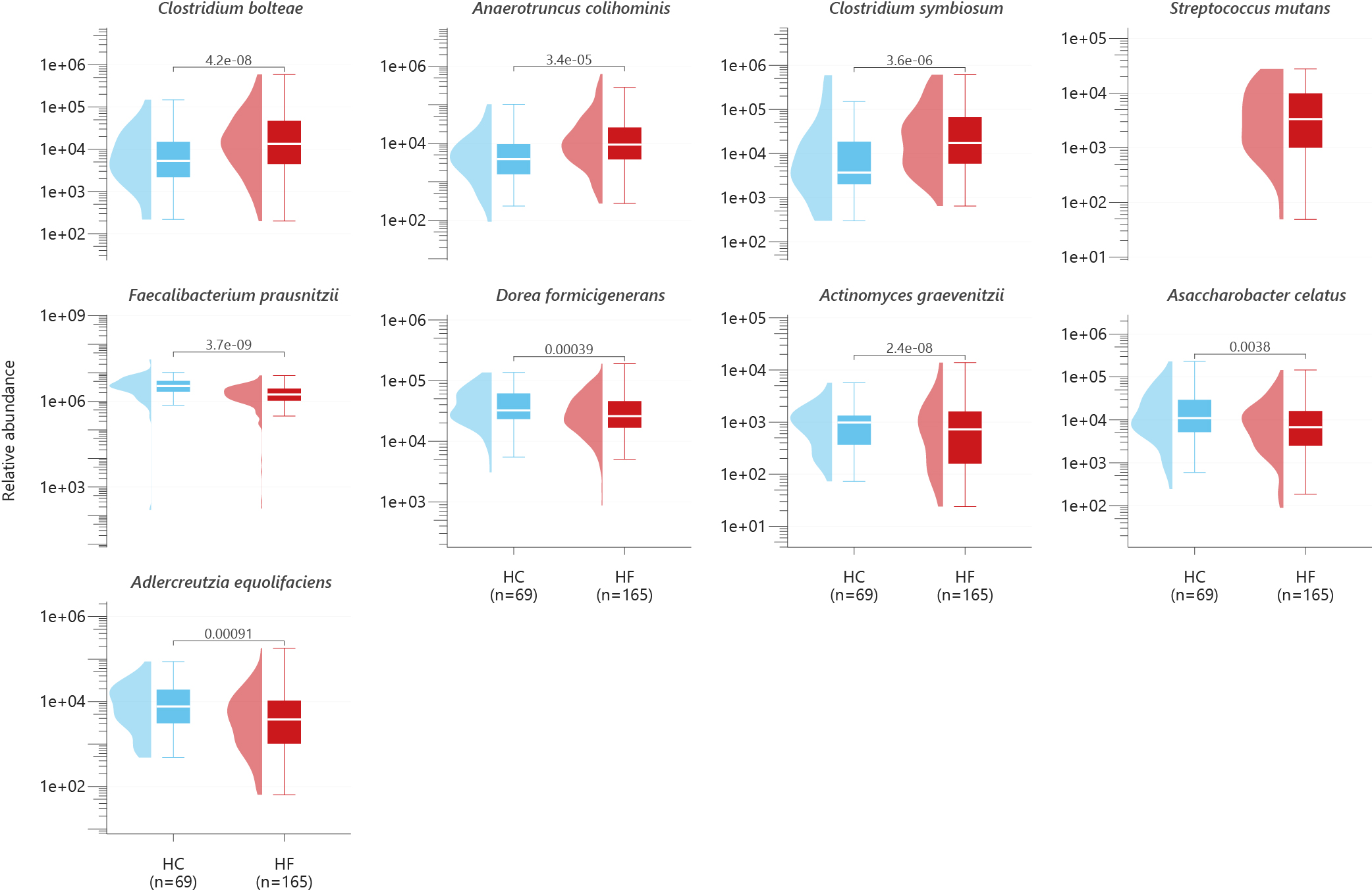


**Fig. S2. Bacterial alteration in heart failure and controls in the total study cohort**. Distributions of differentially abundant bacterial species in heart failure (HF) compared to healthy controls (HC) after adjustment for age, sex and BMI. Data are shown as box- and violin plots. Statistical significance was tested using Wilcoxon tests.

**
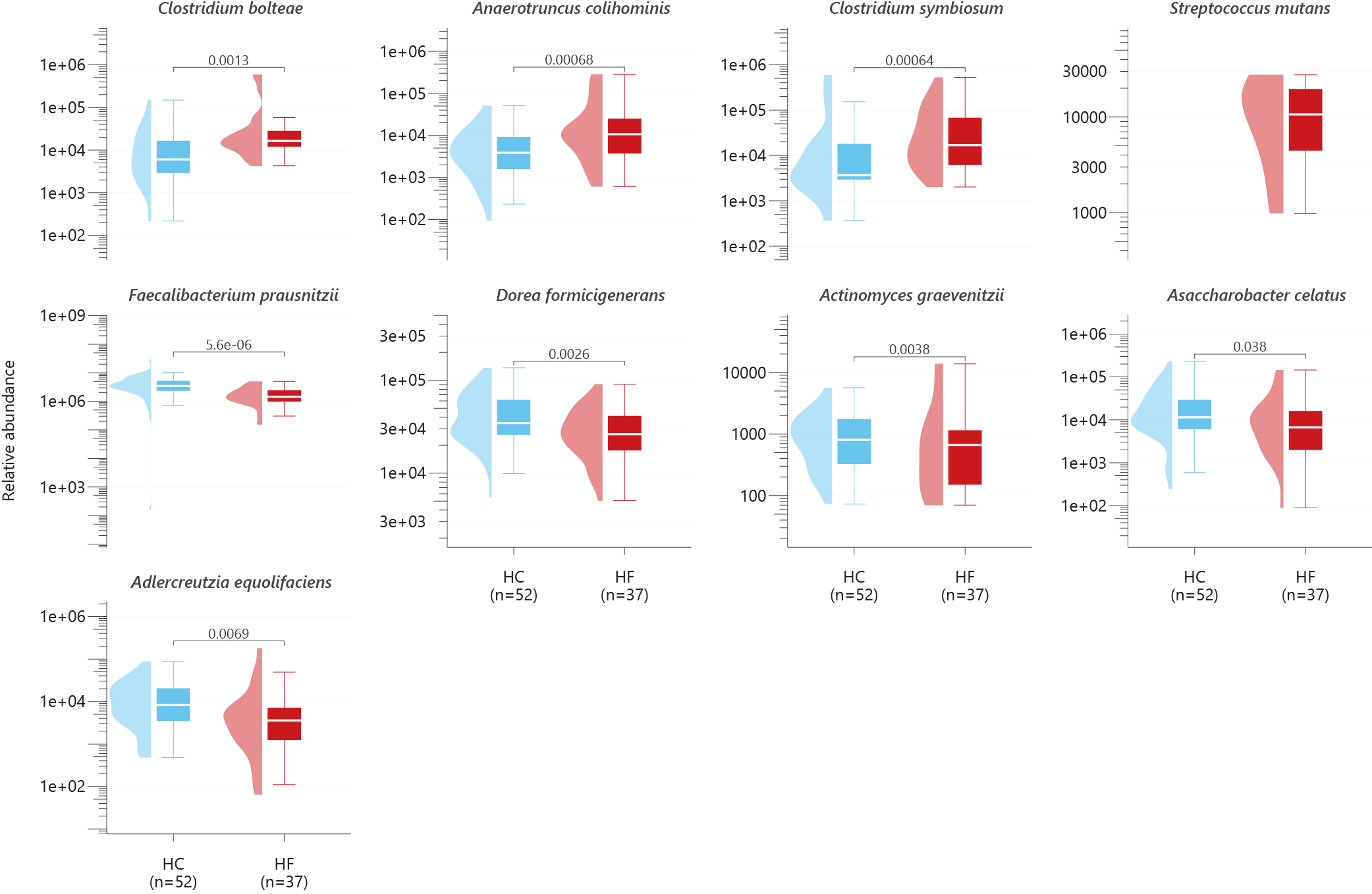
**

**Fig. S3. Bacterial alteration in heart failure and controls in a subsample of study participants matched by age, sex and BMI**. Distributions of differentially abundant bacterial species in heart failure (HF) compared to healthy controls (HC), in a subsample with comparable age (± 1 year) and BMI (± 1 kg/m^2^). Data are shown as box- and violin plots. Statistical significance was tested using Wilcoxon tests. See also additional file 2 table S4.

**
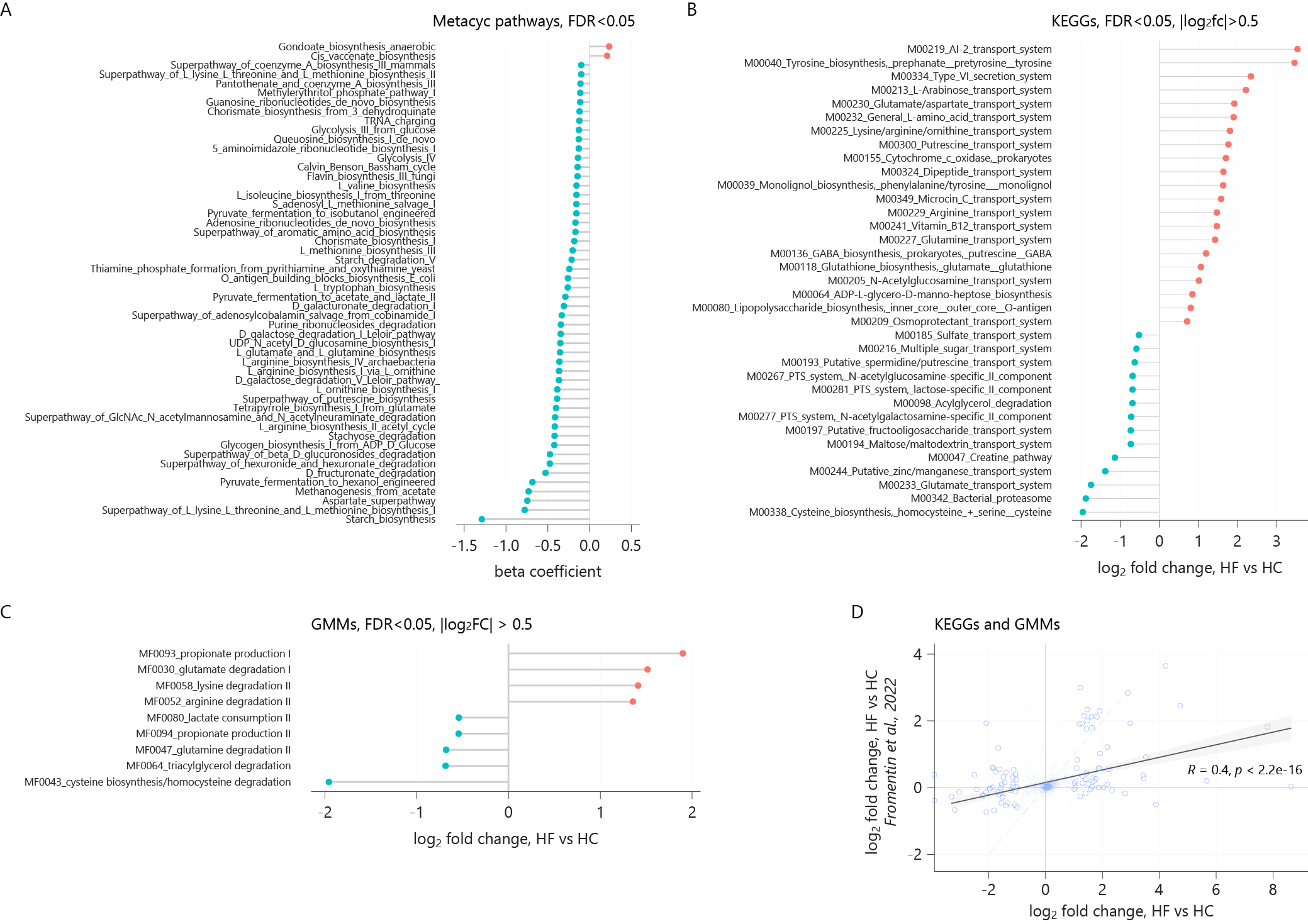
**

**Fig. S4. Bacterial function regulated in heart failure.** **A**. Beta coefficients estimated using a multivariable MaAsLin2 analysis, including age, sex and BMI as covariates, of bacterial pathways sourced from the MetaCyc database. The pathway’s association with heart failure [HF, versus healthy controls (HC)] is shown as beta coefficients from the MaAsLin2 analysis. **B-C**. Log_2_ fold changes of bacterial functions according to KEGG and GMM gut metabolic modules of HF versus HC. Only statistically significantly are shown [FDR<0.05 for regulated bacterial functions with log_2_ fold change (HF versus HC) > 0.5] . See also Supplementary table ST6. **D.** Scatterplot of pooled KEGG and GMM bacterial functions from the current study and the MetaCardis study. Functions with |log_2_FC| > 1.0 are emphasized. A linear regression line was fitted to the data, and results from a Spearman’s correlation is shown in the plot. KEGG: metagenome functions based on Kyoto Encyclopedia of Genes and Genomes GMM: gut specific metabolic modules FDR: false discovery rate.


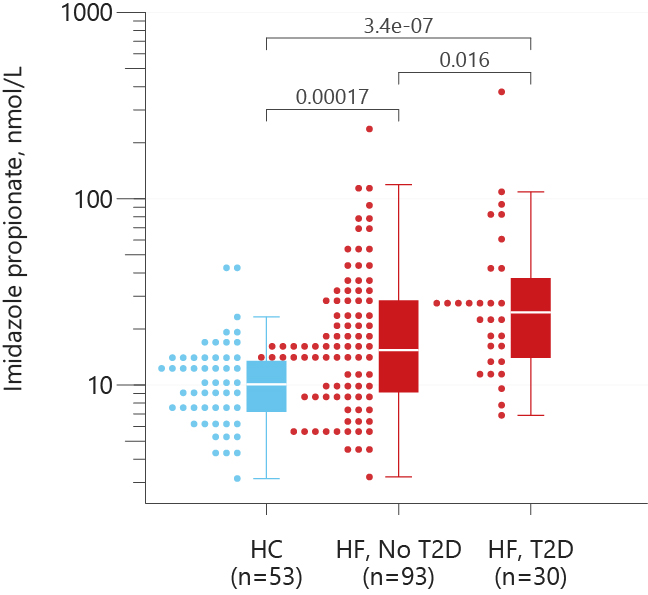


**Fig. S5.** **Imidazole propionate is increased in subjects with type 2 diabetes**. Distributions of imidazole propionate serum levels in healthy controls (HC) and heart failure (HF) patients with or without type 2 diabetes (T2D) are shown as box- and dotplots. Statistical significance was tested using a Wilcoxon test.

***
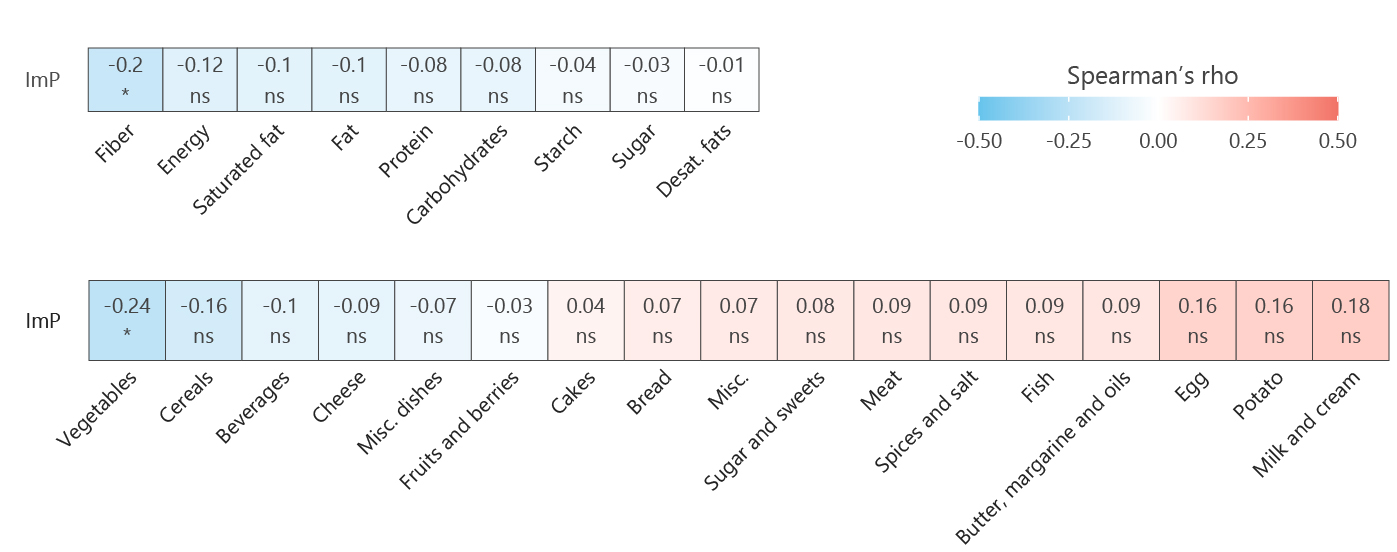
***

**Fig. S6. Imidazole propionate serum levels are associated with an unhealthy diet in subjects with heart failure. A-B.** Correlation matrix for imidazole propionate serum levels and macronutrients and food categories in subjects with heart failure. Spearman’s correlation was calculated for ImP versus each macronutrient and food category. ^*^: p < 0.05; ns: p > 0.05.
